# Nanoscale Surface Metal-Coating Method without Pretreatment for High-Magnification Biological Observation and Applications

**DOI:** 10.3390/biomimetics9100588

**Published:** 2024-09-28

**Authors:** Kenshin Takemura, Taisei Motomura, Yuko Takagi

**Affiliations:** 1 Sensing System Research Center, National Institute of Advanced Industrial Science and Technology (AIST), Tosu 841-0052, Saga, Japan; t.motomura@aist.go.jp; 2 Biomedical Research Institute, National Institute of Advanced Industrial Science and Technology (AIST), Tsukuba 305-8566, Ibaraki, Japan; yuko-takagi@aist.go.jp

**Keywords:** bioimaging, metal thin film, sputtering, surface analysis

## Abstract

Biospecimen imaging is essential across various fields. In particular, a considerable amount of research has focused on developing pretreatment techniques, ranging from freeze-drying to the use of highly conductive polymers, and on advancements in instrumentation, such as cryogenic electron microscopy. These specialized techniques and equipment have facilitated nanoscale and microscale bioimaging. However, user access to these environments remains limited. This study introduced a novel technique to achieve high conductivity in bioimaging by employing a magnetically controlled sputtering cathode to facilitate low-temperature deposition and low-electron bombardment. This approach allows for the convenient high-magnification observation of highly structured three-dimensional specimens, such as pill bugs and butterfly wings, and fragile specimens, such as single-cell protozoan parasites, using metal deposition only. Furthermore, it is easily accessible in the field of bioimaging because it does not require any pretreatment and enables surface analysis of biospecimens with an electron microscope using only a single pretreatment process. Protozoa, which are microorganisms, were successfully observed at high magnification without structural changes due to thermal denaturation. Furthermore, metallic film deposition and electrochemical signal measurements using these metallic films were achieved in pill bugs.

## 1. Introduction

Metal coatings are extensively used in bioimaging to impart conductivity to objects [1]. This protects the sample from potential damage caused by beam irradiation in scanning electron microscopy (SEM) and transmission electron microscopy [2]. Although aluminum, gold, and carbon are commonly deposited via vapor deposition techniques [3,4], biomaterials can undergo shape changes owing to thermal denaturation and rapid dehydration during metal deposition without pretreatment [5]. High-magnification observation is necessary in the field of morphology to observe the original shape and perform scientific analysis. In cryogenic SEM (cryo-SEM), samples are rapidly frozen and maintained at ultralow temperatures to enable detailed surface imaging using a scanning electron beam [6,7]. Other techniques, such as crushing during observation, have been implemented to confirm both the structure and functionality of the sample [8]. Furthermore, the observation of living organisms has been reported using “nanosuit” technology, which involves coating with conductive polymers to prevent moisture evaporation during pretreatment [9,10]. However, these techniques present challenges when analyzing the same specimen with multiple instruments. Therefore, it is beneficial to coat biological specimens with metallic materials that are resistant to oxidation and cause minimal damage.

Uniform high-quality metal deposition is essential for accurate observations, with magnetron sputtering being the ideal deposition method [11,12]. During the deposition process, the secondary electrons generated from the target material surface accelerate the electric field, which in turn causes a sputtering effect by the ions in the plasma. By providing conductivity for imaging purposes, the deposited metal can increase the sample temperature more than metals coated by vapor deposition methods [13]. However, plasma collision is inevitable in magnetron sputtering, leading to the decomposition of interfacial molecules and structural changes that ultimately affect the observation [14,15]. Moreover, the radicals generated during magnetron sputtering are extremely harmful, such that they can be used for sterilization, regardless of the gas species used (oxygen or argon) [16]. Thus, this method is unsuitable for observing and analyzing biological samples that require conditions that resemble those of their natural environment. Low-temperature magnetron sputtering techniques have been documented, but their effectiveness varies depending on the equipment configuration and arrangement of the magnet and cathode body [17,18]. Nonetheless, in all equipment configurations, plasma impingement on the sample is unavoidable. Moreover, this technique has yet to be established as a low-damage deposition method for imaging purposes.

**Figure 1:**
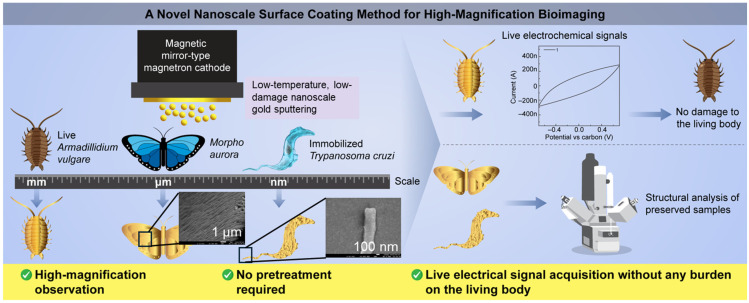
Schematic of nanoscale surface metal-coating method and bioimaging applications.

In this study, low-damage bioimaging was conducted on different samples, including the nanostructured to microstructured pill bug exoskeleton as a vacuum-resistant biological sample, a morpho butterfly wing known for its complex three-dimensional (3D) microstructure, and a protozoan parasite as a unicellular organism without rigid extracellular substances (Figure 1). Gold, which has excellent electrical conductivity and oxidation resistance, was selected as the material for the conductive film coating. The deposition density of the 3D structures and their effectiveness in high-magnification observations were investigated. Furthermore, potential applications of the low-damage deposited metal films on biological samples were explored using a live pill bug.

## 2. Materials and Methods

### 2.1. Materials and Instrumentation

A QUICK COATER SC-701HMC II (Sanyu Electron, Tokyo, Japan) was employed as the magnetron sputtering system for comparison. Laser microscopy was conducted using a VK-X3000 microscope (Keyence, Osaka, Japan). SEM was performed using JSM-9100F (JEOL Ltd., Tokyo, Japan) and Ultra Plus (Carl Zeiss Microscopy GmbH, Jena, Germany) instruments. Energy-dispersive X-ray spectroscopy (EDS) was performed using a spectrometer (Oxford Instruments, Abingdon-on-Thames, UK) equipped with an XFlash 6-30 detector (Bruker Nano GmbH, Berlin, Germany). The nanoscale surface roughness and height were assessed using atomic force microscopy (AFM; MFP-3D Origin+, Oxford Instruments, Abingdon, UK). Electrochemical measurements were conducted using an electrochemical analyzer (ALS 630C, BAS Inc., Tokyo, Japan).

### 2.2. Biological Samples

The *Morpho aurora* was purchased from the online shopping site Nature shop (Japan). A section of the wing was severed as a specimen, and was affixed to the substrate using a piece of tape. The *Trypanosoma cruzi* was cultured alongside host Swiss 3T3 cells in Dulbecco’s Modified Eagle’s medium containing 10% fetal bovine serum (FBS) at 37 °C in a CO_2_ incubator. The trypomastigote stage of *T. cruzi* (Trypo) was separated from the supernatant of the host–parasite coculture by centrifuging the mixture of Trypo and host cell debris and allowing active Trypo to swim out of the resulting pellet [19]. The epimastigote (Epi) stage was cultured axenically at 27 °C in liver-infusion tryptose medium with 10% FBS. The collected *T. cruzi* was fixed with formalin and stored in phosphate-buffered saline until use. *Armadillidium vulgare* (pill bug) specimens were reared in boxes filled with humus for insect breeding. The *A. vulgare* specimens were immobilized using a piece of tape during deposition.

### 2.3. Gold Sputtering on Biological Samples

Low-temperature sputtering was conducted using a magnetic mirror-type magnetron cathode (M3C), which enables low-temperature sputtering deposition at approximately 40 °C by effectively confining plasma effects above the target material [20,21]. The magnets on the cathode are designed to trap the accelerated electrons emitted from the target to prevent the rapid heating of the sample. This process involved the application of radio frequency power of 30 W at 13.56 MHz. The process chamber was evacuated using a turbomolecular pump series, and the base pressure was maintained at less than 2 × 10^−4^ Pa before introducing the samples. Once the biological samples were introduced into the chamber, the moisture content within them increased the base pressure to 2 × 10^−2^ Pa. The argon gas pressure and flow rate were maintained at 0.13 Pa and 10.0 sccm, respectively. The gold target used in the deposition process had a purity of 99.99%, diameter of 50 mm, and thickness of 2.5 mm. The distance between the target and substrate surface remained fixed at 50 mm, and the deposition time was 10 min. During the gold sputtering process, the samples underwent vacuum and plasma treatment for 15 min.

### 2.4. Bioimaging of Sputtered Samples 

**Figure 2:**
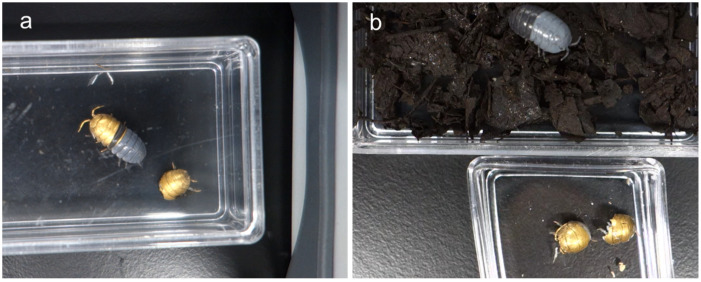
Images of (**a**) half-molted and (**b**) fully molted *A. vulgare* specimens.

The biological samples subjected to metal deposition were observed using laser microscopy and SEM at a high magnification under high vacuum conditions. The morphologies of the microstructures on the *M. aurora* wing surfaces were examined in detail. In addition, the surface morphology of the protozoa was observed using the Trypo and Epi stages of *T. cruzi*. The *A. vulgare* specimens lived and molted at 3 weeks without any issues in their biological conditions after deposition. SEM was conducted to examine the surface of their exoskeletons after molting (Figure 2a,b).

### 2.5. Electrochemical Monitoring of A. vulgare Using Sputtered Gold Film

The *A. vulgare* specimens were gold-plated while alive, providing their shells with conductive surfaces. Deposited individuals for bioimaging were kept under the appropriate conditions until molting. One specimen that was not used for the observation was selected to obtain biological signals in a living state using the deposited gold film. Electrochemical measurements were conducted by attaching two conductive carbon tapes to the surface as the working and counter electrodes, with the surface gold film serving as the individual electrolyte. The pill bugs were burrowing in the soil at the time of electrochemical measurements. The attached carbon tapes served as reference electrodes. Cyclic voltammetry (CV) and differential pulse voltammetry (DPV) were performed. Fifty cycles of CV were performed at a voltage sweep rate of 100 mV/s from −0.8 V to 0.8 V. DPV was used to measure the aromatic compounds by sweeping the voltage from 0.8 V to 1.2 V, with the current peak indicating the oxidation of hydroxyl groups in these aromatic compounds. 

## 3. Results and Discussion

### 3.1. Detail of Deposited Gold Nanoparticles

**Figure 3:**
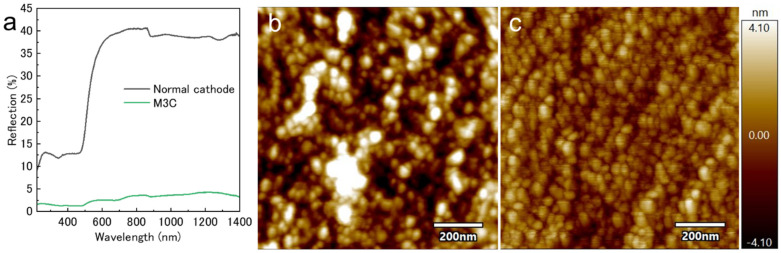
(**a**) Reflectance measurements of the gold-plated glass substrates. AFM images of a gold thin film interface deposited using (**b**) conventional magnetron sputtering and (**c**) M3C.

The surface reflectance of a gold film deposited on a commercial glass slide using either a conventional magnetron sputtering system or an M3C was measured. The gold sample deposited with the conventional cathode exhibited higher reflectance at higher wavelengths, which is consistent with the reflectance spectral features of bulk gold thin films deposited under high vacuum [22]. In contrast, only the M3C-deposited sample exhibited a strong absorbance at approximately 500 nm, which is characteristic of gold nanoparticles (Figure 3a). The volume fraction of gold nanoparticles correlates with the redshift of the reflection spectrum, with increased reflection at longer wavelengths for films deposited at low temperatures [23]. Both samples exhibited a bulk gold-like color and a mirror-like surface. The optical properties indicate that the low-temperature-deposited sample comprised nanomaterials. Furthermore, AFM was employed for the surface analysis to examine the interface structure after deposition (Figure 3b,c). The surface roughness of both samples was at the nanoscale, reflecting the smoothness of the glass surface of the deposited substrate. During gold deposition using a conventional cathode, grains larger than 100 nm were randomly formed on the surface alongside a particulate structure with a size in the range of 10–20 nm. In contrast, the particle diameter during low-temperature deposition did not vary significantly, i.e., the particle diameters were less than 100 nm. Both the optical and surface analysis results suggest that bulkification due to crystal growth did not occur in the gold films formed by low-temperature deposition. When a metal undergoes crystal growth, a bulk material is formed from the starting point particles covering the surface of the sample. When applied to bioimaging, the deposition time must be optimized to avoid disturbing the observations. By contrast, the present deposition method, in which nanoparticles are stacked, did not damage the shape of the sample; therefore, it was easy to set the parameters.

### 3.2. Sputtering on M. aurora 

**Figure 4:**
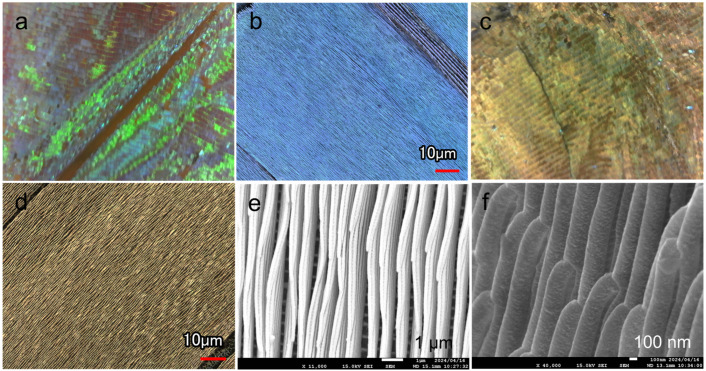
(**a**,**c**) Optical and (**b**,**d**) laser microscopy images of the wing surface (**a**,**b**) before and (**c**,**d**) after gold deposition. (**e**) Top-view SEM image of the wing surface microstructure after deposition. (**f**) Side-view image showing the stacked microstructure of the sample in (**e**).

Gold was deposited on *M. aurora* wings, which exhibit structural coloration under polarized light conditions, followed by observation. Before deposition, the specimens exhibited a bright blue color, as shown in the optical and laser microscopy images (Figure 4a,b, and Figure S1A). Their surface features numerous grooves with wall-like microstructures and uneven wall surfaces, which reflect light from the outside light source and display color from any orientation [24,25]. The optical image had a shiny blue color owing to light reflection. Laser microscopy revealed a bright blue color and numerous longitudinal stripes, which are characteristic of the wing structures of morpho butterflies. Similarly, the gold-deposited wings retained their scales as microwalls (Figure 4c,d), indicating that low-temperature deposition using M3C had no detrimental effects on the observation, such as bulk formation or the covering of scales by crystal growth. However, they did not exhibit an overall blue coloration owing to light reflection. Some regions exhibited a partially blue hue, which can be attributed to alterations in the distance and thickness of the walls owing to the deposition of gold at a thickness of 100 nm in microscopic regions, such as the scale structures. 

The SEM observations of the deposited samples revealed that the wing scales did not detach, and conductivity was imparted (Figure S1). Ridges and crossribs were arranged in a bridge-like structure to support the microwalls on the scale surface (Figure S2). These microscale grids were also visible behind the wall-like lamellae (Figure 4e), suggesting that the gold reached large depths into the complex structure, even in a magnetic-field-controlled environment. Furthermore, side-view observations of the deposited sample revealed the layered stack of scale lamellae in the linear structures (approximate thickness of 200 nm) that resulted in a superhydrophobic structure [26] (Figure 4f). Thin grooves were visible between the layers, indicating that particles can be deposited in the shaded areas of the 3D structure with a certain degree of wraparound motion. 

**Figure 5:**
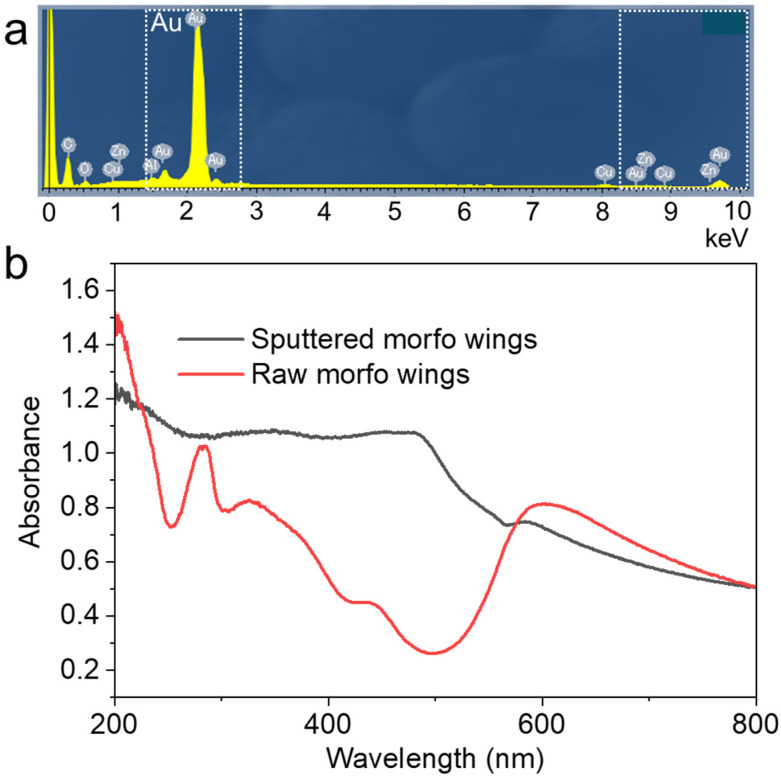
(**a**) EDS elemental analysis of *M*. *aurora* after deposition. (**b**) Measured absorbances of *M. aurora* before and after deposition.

Elemental analysis was conducted on the surface by EDS to verify that the observed image was that of the deposited sample (Figure 5a). Gold exhibited the strongest peak in the elemental analysis. Carbon, copper, and zinc were also detected. The presence of carbon can likely be attributed to the thinness of the gold film, which was only 100 nm; therefore, elements from the morpho butterfly itself were observed. The optical properties of the butterfly wings were also assessed (Figure 5b). The wings, prior to deposition, exhibited an absorbance peak at 280 nm, indicating their protein composition [27,28]. The absorbance was lower in the range of 400–500 nm, suggesting the reflection of blue wavelengths [29]. After deposition, blue wavelengths were no longer reflected, and an absorbance peak at 500 nm, characteristic of gold nanoparticles, was observed [30]. Light must be reflected in the wavelength range of 400–500 nm to attain structural color; however, the strong light absorption of the gold nanoparticles before light entered the structure suggests that the film is unlikely to appear blue after deposition. As the purpose of this study is to observe, it was not assumed that the optical functionality would be maintained. However, functionality can be maintained by ensuring that the deposited metal is less than 100 nm thick and has insufficient blue-light absorbance. 

### 3.3. Sputtering on T. cruzi

**Figure 6:**
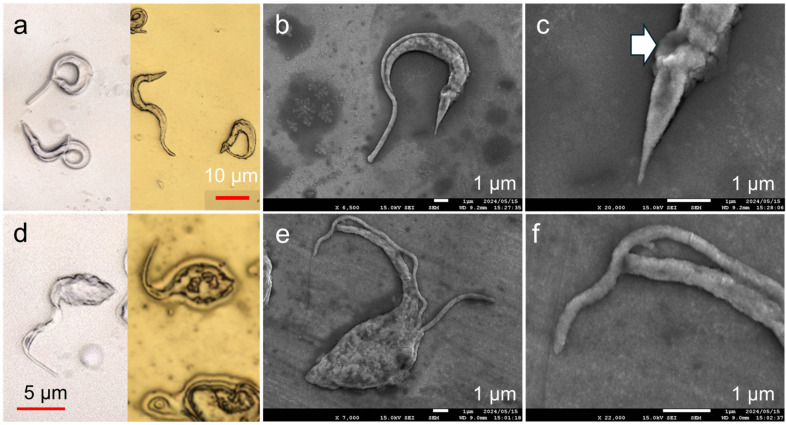
(**a**) Laser microscopy image of Trypo before (**left**) and after (**right**) deposition. (**b**,**c**) High-magnification SEM images of Trypo after gold deposition using M3C. White arrow indicates a dent. (**d**) Laser microscopy image of Epi before (**left**) and after (**right**) deposition. (**e**,**f**) High-magnification SEM image on gold-deposited Epi with a glass surface, which thickened the deposited gold film.

Gold deposition was performed on *T. cruzi* to demonstrate imaging not only on dry-state samples but also on fixed and preserved water-rich microbial samples. The interface was imaged using laser microscopy before and after low-temperature deposition on Trypo (Figure 6a). No structural deformations were observed after deposition from the tip of the flagellum to the bumpy appearance of the kinetoplast. This suggests that gold deposition occurred without affecting the surface structure of the formalin-treated protozoa. The effectiveness of low-temperature deposition on biological samples for observation testing was confirmed by examining the gold-deposited Trypo using high-magnification SEM as a visualization method (Figure 6b). A flagellum observed as a fin-like edge in the laser microscopy image can be observed as a smooth rod along the cell body in the SEM image. The texture of the flagellum is distinct from that of the cell body, suggesting that the roughness on the cell body is not ascribed to the grain formation of gold nanoparticles but actually reflects the cell texture itself. The posterior tip of the cell had dimensions in the order of nanometers and was thinner than the tip of the flagellum (Figure 6c). The dent near the bumpy kinetoplast likely marks the position of the flagellar pocket, or possibly reflects a minor damage caused by the coating process (Figure 6c, arrow) 

Similar tests were conducted on the Epi stage to demonstrate the effectiveness of low-temperature metal deposition in observing the structural differences of *T. cruzi* at different developmental stages (Figure 6d). Consequently, the tip of the gold-deposited flagella, which could not be observed well before coating, was successfully imaged. No significant shape change was observed before and after deposition. Similar to Trypo, the low-temperature process did not significantly affect the shape of the cell body. The high-magnification image after gold deposition shows that *T. cruzi* has one flagellum, whereas some Epi cells have two flagella, representing the growth process in preparation for cell division (Figure 6e). Focusing on the flagellum tip, even the nanoscale region was evenly deposited for observations (Figure 6f). This indicates that gold deposition was adequately mild to preserve the delicate cellular structure, even though *T. cruzi* does not possess rigid extracellular support, unlike insects, which possess a well known sturdy material called chitin. The changes in the surface morphology over time owing to sample preparation techniques, such as lyophilization, for high-magnification observation pose a challenge in the observation of biological samples. As such, low-temperature processes can be employed to address this issue. To demonstrate its reproducibility, this low-damage, low-temperature sputtering was performed on three adsorbed Trypo and Epi substrates fabricated under the same conditions (Figure S3). Because sputtering was used, a large area of the sample could be coated. The protozoa on each of the three substrates showed no significant differences in their surface morphology, indicating high reproducibility.

### 3.4. Sputtering on Living A. vulgare 

**Figure 7:**
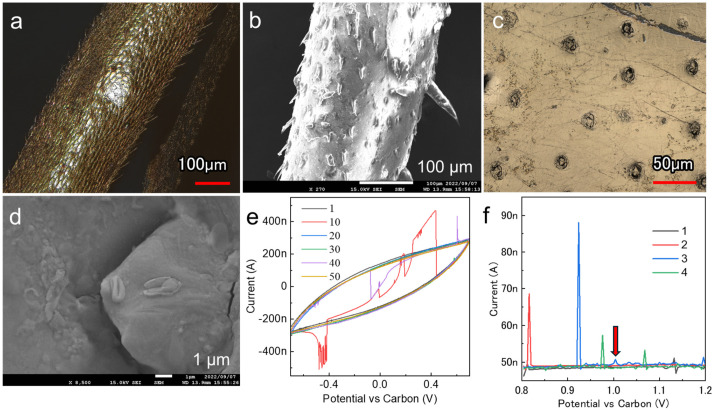
(**a**) Laser microscopy image of a leg after deposition. (**b**) High-magnification SEM images of a molted leg. (**c**) Laser microscopy images of the pore channels on the exoskeleton surface. (**d**) High-magnification image of the spiracle of the exoskeleton after molting. Electrochemical measurements obtained using (**e**) CV and (**f**) DPV.

The pill bug was immobilized facing the cathode, and gold was deposited on all parts of the body, except for the area covered by the tape used for immobilization. Laser microscopy revealed that particle wraparounds caused by gold nanoparticle deposition covered the legs (Figure 7a). The exoskeleton, together with the gold film, was molted from the body surface at the rear. Further high-magnification observation of the molted legs revealed microscopic thorn-like structures growing on the surface (Figure 7b). This suggests that the gold nanoparticles emitted from the cathode during deposition fully reached the sample surface, wrapped around the 3D structure, and deposited on the sample surface. Numerous pore channels were observed on the exoskeleton surface. Laser microscopy images show that the pores were entirely gold-colored, suggesting that conductivity was imparted successfully (Figure 7c). Even the smallest pores were not blocked by the film deposition process (Figure 7d). The formation of the nanoparticle film achieved by low-temperature deposition was adaptable to all material types with a low thermal tolerance, from nanoscale to macroscale structures. Notably, pill bugs have an exoskeleton and are resistant to evaporation, facilitating their survival after deposition [31,32]. Therefore, this technique can be used for observation purposes and other applications.

### 3.5. Applications of Gold Film Deposition beyond Imaging

Electrochemical analyses were conducted on living pill bugs with a gold film at the interface. Fifty CV cycles were performed to demonstrate the possibility of monitoring electrochemical reactions over time using the deposited gold thin films as individual electrolytes (Figure 7e). As the reference electrode was made of carbon tape, a discrepancy between the measured and actual sweep voltages was assumed; however, we successfully recorded continuous currents at the nanoampere level. The signal followed a simple CV profile with numerous instantaneous blips in the current values, corresponding to the movement of individual samples attached to the electrode. Although capturing random movements of pill bugs in soil using optical applications is challenging, applying voltage to the electrodes facilitated the monitoring of their activity levels. This possibility was tested using DPV, which is a highly sensitive electrochemical measurement technique (Figure 7f). The current increased almost instantaneously in response to the movement, as observed by CV. In the third measurement, a current peak with a wide half-width of approximately 1 V was observed (arrow), which can be ascribed to the gaseous components adhering to the carbon tape through the pores and an oxidation response owing to the electron exchange through the gold electrolyte. As insects with exoskeletons have water-repellent surfaces, commonly used measurement methods, such as piercing the body with electrodes, can significantly affect the living organism [33]. We successfully acquired electrochemical signals without interfering with the natural movement of the soil-dwelling organisms by treating the deposited gold as an individual electrolyte in the experimental setup. The number of samples on which live metals could be deposited was limited. In contrast, low-temperature deposition provides new signals from previously unexplored perspectives. By selecting the optimal deposition conditions and electrode materials, the type of gas released from the pore channel could be monitored and identified to achieve real-time biomeasurements by minimally invasive electrochemical detection. 

This technique for monitoring insects is compared with previously reported ones in Table 1. An advantage of using electrodes is that the movement of an individual can be quantified as an electrical signal. Until now, the most common method has been to implant shadow rod-shaped electrodes in the living body. By using a nearly weightless thin film and directly using the increase or decrease in the current value due to vibration from the wing as a signal, a more accurate motion sensor could be achieved. Acoustic and laser sensors, which are capable of large-scale environmental sensing, have strengths in group analysis and are used to generate data on seasonal group movements. However, ecological monitoring focusing on specific individuals within a population is difficult. In addition, neither technique is suitable for insects that burrow and hide in soil. Small devices that can remotely acquire electrical signals have been developed and can be combined with thin metal films to quantify the signals of insects moving in the soil. This is likely to add new knowledge to the existing monitoring technologies.

Compared with previous techniques for insect biomonitoring, the method in this study is significantly less invasive (Table 1). In addition, it is possible to design and attach a flexible protective layer in advance, such as a circuit, to form an electrode layer that is more suitable for measurement. In combination with a small Bluetooth module, signals can be acquired remotely without excessive interference from the movements of the insect’s body.

The results suggest that a metal deposition method that uses magnetic field control to achieve low temperatures has excellent low-damage performance for biosamples. Lyophilization, the most versatile pretreatment that has been used for imaging biomaterials, is an excellent preservation technique. However, shape changes due to processing have become an issue (Table 2). The protection of biospecimens by freeze-drying enables high-magnification observations using metal deposition. However, the resulting structural deformation of the samples has not yet been resolved. Research on conductive polymers has progressed as a method for protecting samples from vacuum conditions without drying. In addition, tardigrades have been observed while still alive using a remarkable technique that does not damage biospecimens. Meanwhile, polymer coatings are often polymerized by X-ray irradiation. Although this irradiation time is extremely short, the effect of the X-rays on minute biospecimens can be significant. Resin materials are the most suitable when focusing only on high-magnification observations. By contrast, low-temperature metal deposition allows the analysis of both electrical and optical signals from the deposited metallic films. Thus, low-temperature deposition is the most multifaceted evaluation method for the pretreatment of biosamples. 

To demonstrate the versatility of this technology, low-temperature deposition was applied to lotus leaves, a biomimetic material. No changes were observed on the surface before and after deposition. The short exposure time to vacuum and the ability to turn on the plasma and quickly deposit a metal film on the sample, even if moisture occurs over time, demonstrate the high applicability of this technology (Figure S4).

## 4. Conclusions

To effectively observe biological structures, an optimal pretreatment process is needed to protect objects from thermal damage and the strong oxidation effects of plasma, while preventing changes in the interface structure due to particle growth during the magnetron sputtering of metal films. This study demonstrated that low-temperature deposition using M3C addressed these challenges and facilitated high-magnification observation using metal deposition without any pretreatment. This deposition method, in which plasma collisions are suppressed by magnetic field lines, succeeded in imparting conductivity through metal deposition that was sufficient for observation, even on complex structures, such as butterfly wings. Furthermore, microorganisms, such as protozoa, could be observed without destroying their structures. When the film was deposited on protozoa, which are microorganisms, the surface morphology was preserved after deposition, even when the tests were repeated. The containment of accelerated electrons by magnetic field control is effective for the bioimaging applications of metal film deposition. 

A gold film was successfully deposited on *A. vulgare* and exhibited durability against evaporation under certain moisture conditions. After gold deposition, *A. vulgare* survived without issues, and the deposited shell structure remained intact until the old exoskeleton was removed via molting. These results suggest that nanoparticle deposition via low-temperature techniques achieved good film adhesion and resistance to physical friction, such as burrowing into the soil. Leveraging this property, electrochemical measurements using the gold film as an electrolyte successfully detected a current increase corresponding to the movement and an electrochemical oxidation peak corresponding to a gas component via DPV. To date, live electrical signals have primarily been acquired by inserting electrodes into the body; however, our technique was noninvasive in living organisms. Moreover, this method can be used to analyze the functions of uncharacterized organisms. In the future, we will conduct electrochemical analyses of living organisms using composite materials, such as platinum and MoS, which have strong electrochemical and catalytic activities, to explore a more diverse range of species.

## Figures and Tables

**Table 1:** Comparison of insect monitoring techniques.

Sensor Material	Signal	Sample Number/Sensor	Information	Invasiveness	Ref.
Implanted electrode	Electric	Individual	Movement	High	[34]
Acoustic sensor	Acoustic	Group	Sounds	-	[35]
Radar detector	Optical	Group	Size, shape, speed	-	[36]
Surface electrode	Electric	Individual	Movement, solution from the insect’s body	Low	This work

**Table 2:** Comparison of low-temperature deposition with other reported pretreatments for biosamples.

Pretreatment	Advantage	Disadvantage	Ref.
Dry freezing	Long-term stable storage	Long vacuum exposure time, immobilization with protective reagents for drying	[37]
Polymer coating	Protection from desiccation, live observation of specific organisms	Brief X-ray exposure for polymerization	[38]
Metal coating	Application analysis using metallic films	Brief vacuum and plasma exposure, heat damage, lyophilization required	[39]
This method	Live observation of specific organisms, application analysis using metallic films, large-area coating	Brief vacuum exposure	-

## Data Availability

The datasets generated and/or analyzed during the current study are available from the corresponding author upon reasonable request.

## Supplementary Materials

The following supporting information can be downloaded at https://www.mdpi.com/article/10.3390/biomimetics9100588/s1, Figure S1: (A) Wings of morpho butterfly specimens observed by laser microscopy at a magnification of 20×. (B) Image of the wing surface of a morpho butterfly after deposition observed at 250× by SEM under high-vacuum conditions; Figure S2: Bridge-like microstructure on the morpho wing was observed by scanning electron microscopy; Figure S3: Laser microscopy images of substrate surfaces fabricated to test the reproducibility of low-damage gold deposition using Trypo and Epi; Figure S4: Laser microscopy images of the surface of lotus leaves before and after gold deposition. Movie S1: Video of half-molted *Armadillidium vulgare*.
