# Supplementary material for: Nanoscale Surface Metal-Coating Method without Pretreatment for High-Magnification Biological Observation and Applications
Source: Biomimetics (Basel). 2024 Sep 28;9(10):588. doi: 10.3390/biomimetics9100588 (PMC11504977; doi:10.3390/biomimetics9100588)
Supplement: Supplementary file 1 [file biomimetics-09-00588-s001.zip › biomimetics-3194970-supplementary-english.pdf]

Supplementary Materials for

Nanoscale surface metal-coating method without pretreatment for biological high-magnification observation and applications

Kenshin Takemura[1], Taisei Motomura[1], Yuko Takagi[2]*

[1]Sensing System Research Center, National Institute of Advanced Industrial Science and Technology (AIST), Tosu, Saga, Japan

[2]Biomedical Research Institute, National Institute of Advanced Industrial Science and Technology (AIST), Tsukuba, Ibaraki, Japan

Corresponding author: takemura.kenshin@aist.go.jp

The PDF file includes:

Figures S1 to S4

Movie S1

Figure S1. A, B

(A) Wings of morpho butterfly specimens observed by laser microscopy at a magnification of 20x. (B) Image of the wing surface of a morpho butterfly after deposition observed at 250x magnification by SEM under high-vacuum conditions.

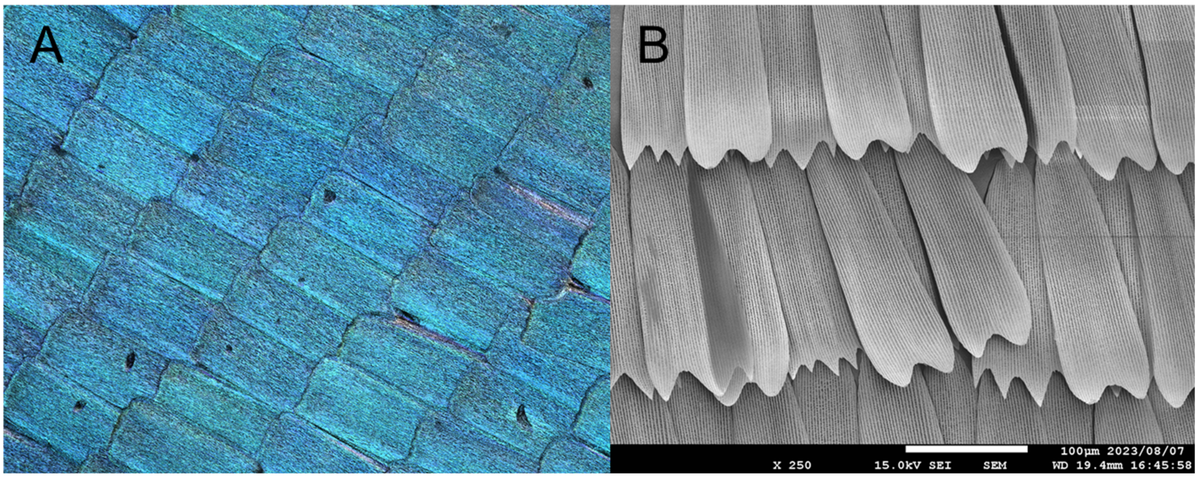

Figure S2.
Bridge-like microstructure on the morpho wing was observed by scanning electron microscopy.

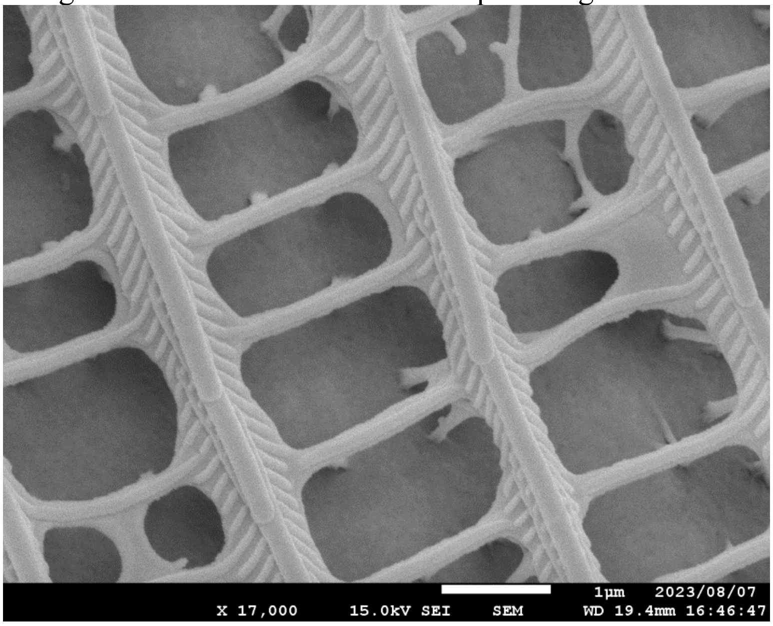

Figure S3.
Laser microscopy images of substrate surfaces fabricated to test the reproducibility of low-damage gold deposition using Trypo and Epi.

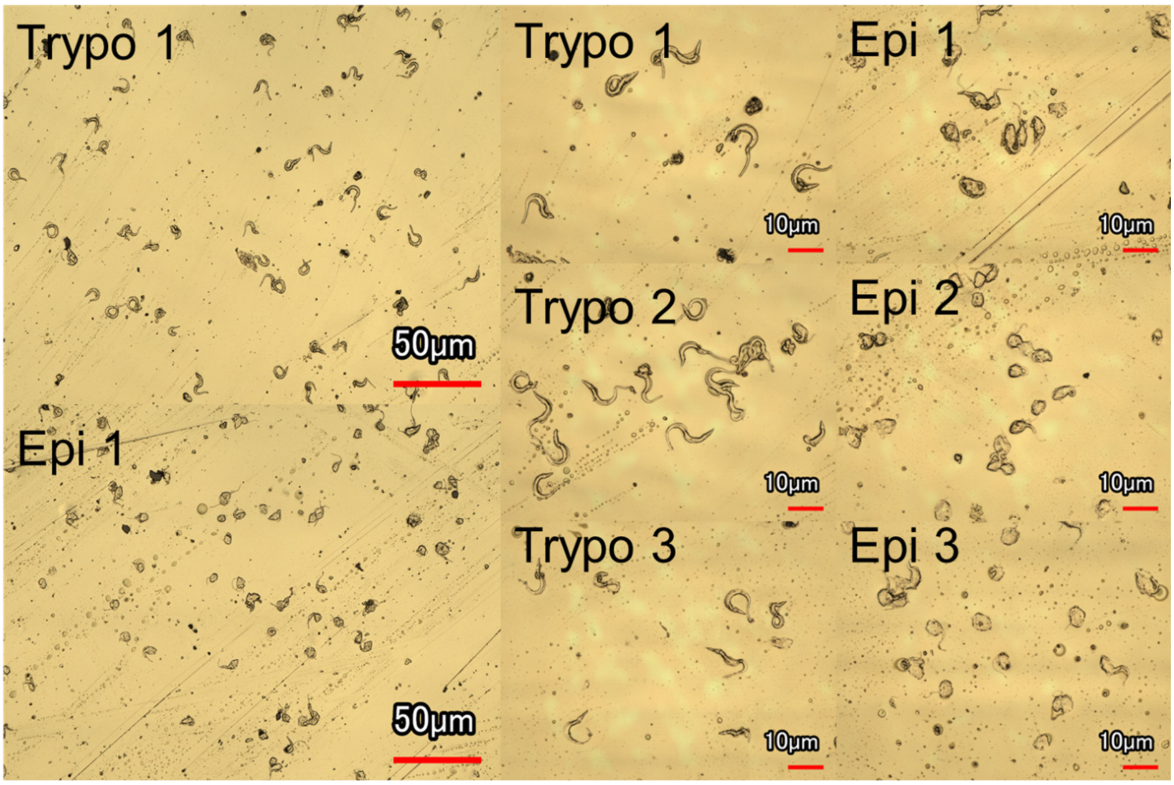

Figure S4.
Laser microscopy images of the surface of the lotus leaves before and after gold deposition.

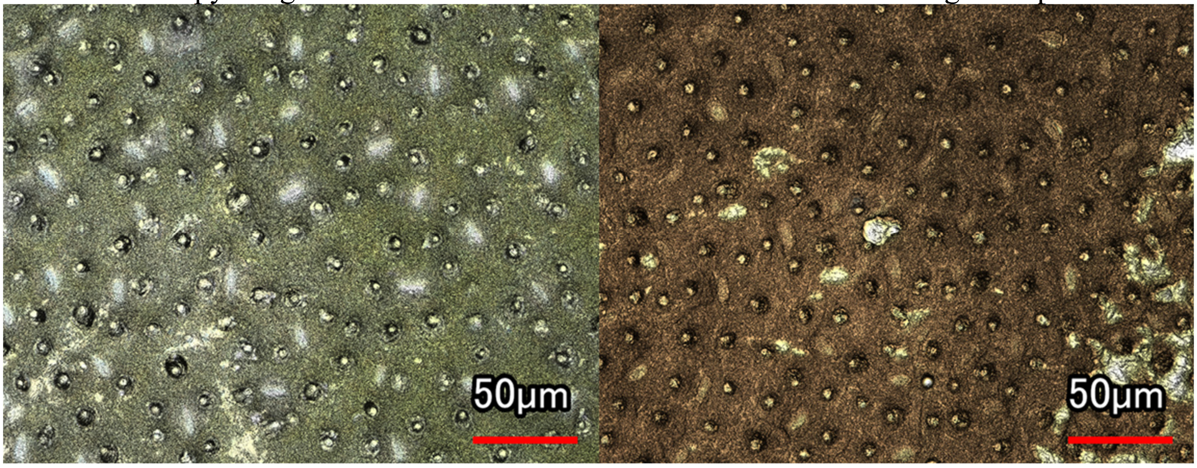

Movie S1.

Video of half-molted *Armadillidium vulgare.*
